# Emile Zola as a Degenerate

**Published:** 1897-04

**Authors:** 


### EMILE ZOLA AS A DEGENERATE.

Whatever may be the view held as to the merit of Nordau's "Degeneration," it has widened to a large extent the discussion of the subject and focused attention on the principles advanced by Lombroso and his school. The charge made by Nordau, that Zola exhibits the stigmata of degeneration, has been taken up by a French physician, Dr. Toulouse, who is chief of clinic for mental diseases at the Medical Faculty of Paris, and publishes his results as a medico-physiologic inquiry into the relationship between intellectual superiority and neuropathy. We have not the original essay before us, but an extended criticism of it by Lombroso (*Medical Week*, Jan. 22, 1897).

Dr. Toulouse has conducted his investigation in a strictly scientific manner. He has not indulged in mere invective or ridicule, as have so many of Nordau's critics. Zola's ancestry is carefully studied. He is shown to be of mixed blood and his mother to have been rather neurotic. Zola himself had an attack of ' brain fever" (whatever that may be) in early childhood, which left some defect of speech and muscular co-ordination. There was early development of sexual impulses. He was not an intelligent schoolboy, having failed in several of his examinations. Incidental to criticism on this point, Lombroso remarks that the conditions of school life are not avorable but inimical to the development of genius. We can agree with him in this. Dr. Toulouse finds that Zola's height is 1.705 meter, while the extreme distance between the tips of the middle fingers is 1.77 meter. In men whose duties involve overstrain of the arms this disproportion might be expected, but in Zola's case it is regarded by Lombroso as a sign of degeneration. Excessive length of the arm is rather frequent in criminals. Zola's face exhibited numerous frontal wrinkles at the age of six years; his ears are sessile and rather long, which are all regarded as signs of degeneration. We pass over numerous data regarding the special senses and take up discussion of Zola's sexual impulses. Here there is an almost brutal frankness of discussion, which must make rather interesting reading for the subject thereof.

Following the line of investigation that has been presented so elaborately by Von Krafft-Ebing, we are informed that sexual psychopathy is clearly marked, evinced by the evolution of erotic images, by odors in general and by the sight of articles of women's clothing. For proof of this, quotations from his novels are given, showing frequent allusions to objects of this class. Lombroso complains that in conducting the investigation in this phase of character, the proper test was not made. The tendency to exaggeration is also regarded as evidence of degeneration. Zola "attributes to lifeless objects, a fantastic life, transforms them into spectres possessing feelings, will, cunning and thought, while human beings are changed into automatons."

We have given here an abstract of some of the points presented by Lombroso in a long article. Only the striking features have been set forth; there are many details. The whole article is interesting, especially as it is a concrete presentation of Lombroso's theories. Even those who entirely disagree with him will admit that his method is essentially scientific, and if genius be correctly defined as "an infinite capacity for taking pains," the workers of the Lombroso school show genius. It would be interesting to know if Lombroso could prove his own degeneration!

One thought suggests itself to us, and that, how far are the stigmata of degeneration common to all men? Will it not be possible to find as many of these evidences associated with orderly, commonplace individuals as with criminals on the one hand and men of mark on the other?—*Philadelphia Polyclinic.*

